# An early parenting intervention focused on enriched parent–child interactions improves effortful control in the early years of school

**DOI:** 10.1111/cdev.14166

**Published:** 2024-10-02

**Authors:** C. Bennett, E. M. Westrupp, S. K. Bennetts, J. Love, N. J. Hackworth, D. Berthelsen, J. M. Nicholson

**Affiliations:** ^1^ Judith Lumley Centre, School of Nursing and Midwifery La Trobe University Bundoora Victoria Australia; ^2^ Centre for Social and Early Emotional Development, School of Psychology Deakin University Geelong Victoria Australia; ^3^ Murdoch Children's Research Institute Melbourne Victoria Australia; ^4^ Parenting Research Centre Melbourne Victoria Australia; ^5^ School of Early Childhood & Inclusive Education Queensland University of Technology Brisbane Queensland Australia

## Abstract

This study examined long‐term mediating effects of the *smalltalk* parenting intervention on children's effortful control at school age (7.5 years; 2016–2018). In 2010–2012, parents (96% female) of toddlers (*N* = 1201; aged 12–36 months; 52% female) were randomly assigned to either: standard playgroup, *smalltalk* playgroup (group‐only), or *smalltalk* playgroup with additional home coaching (*smalltalk plus*). Multi‐informant data indicated that *smalltalk plus* had unique indirect effects on children's effortful control, through parents' capacity to ‘maintain and extend’ children's focus during joint interactions. Possible mediating pathways via parent verbal responsivity, home learning activities, and descriptive language use were not supported. When parents received a structured playgroup program with additional home coaching, sustainable benefits were evident in children's self‐regulation, assessed in the early school years.

**Abbreviations**
CFI: comparative fit index
EHLS: Early Home Learning Study
IPCI: Indicator of Parent–Child Interaction
IRSAD: Index of Relative Socioeconomic Advantage and Disadvantage
NIH: National Institutes of Health
PALS: Play and Learning Strategies
PAT: Parents as Teachers
RMSEA: root mean square error of approximation
SDQ: Strengths and Difficulties Questionnaire
SEIFA: Socio‐Economic Indexes for Areas
SEM: structural equation model
SES: socioeconomic status
TLI: Tucker–Lewis index
TMCQ: Temperament in Middle Childhood Questionnaire

Children from socioeconomically disadvantaged backgrounds are less likely than their more advantaged peers to enter school ready to learn, and may lack the self‐regulation skills needed for success in formal learning environments (Baker, 2018; Blair & Raver, 2015). This partly reflects more limited access to resources and opportunities in the early home environment which support the development of these skills. Self‐regulation is multi‐faceted, spanning emotional, cognitive, and behavioral processes (Nigg, 2017). Effortful control is one aspect of self‐regulation that refers to the efficient use of executive attention, including the abilities of voluntarily focusing or shifting attention and inhibiting behavior (Eisenberg et al., 2011; Rothbart et al., 2011). In school contexts, effortful control underpins social and emotional competence, and supports classroom learning (Eisenberg et al., 2010). It has been linked to better social engagement with teachers and peers, fewer behavioral problems, stronger school readiness skills, and greater long‐term academic achievement and social–emotional adjustment (Dindo et al., 2017; Eisenberg et al., 2010; Laible et al., 2016; Valiente et al., 2008, 2011). Early parenting interventions, offered prior to school, present an important opportunity for parents to promote the development of effortful control for children from backgrounds of socioeconomic disadvantage.

Effortful control is partly determined by individual differences in temperament. There is also clear evidence that it is shaped by parenting practices, the home environment, and the child's broader social context (Baker, 2018; Eisenberg et al., 2011; Lengua et al., 2014). A stimulating home environment supports early effortful control (Merz et al., 2014) by providing cognitively rich resources and experiences such as access to learning materials, exposure to complex and varied sensory experiences, and frequent quality caregiver–child interactions (Rosen et al., 2020). Opportunities for play, learning, and verbal interaction with caregivers are important for promoting core features of effortful control including task persistence, inhibitory control, and attention maintenance (Mermelshtine, 2017; Olson et al., 2002; Rosen et al., 2020).

Parents can strengthen their child's effortful control by showing interest and encouragement during play and other everyday activities (Kochanska et al., 2000; Lengua et al., 2021; Neale & Whitebread, 2019; Neppl et al., 2020). Such interactions may include making suggestions, commenting on what the child is doing, and using non‐verbal indicators of enjoyment, which can extend children's engagement and completion of tasks. Greater dyadic connectedness (i.e., shared pleasure, mutual enjoyment, conversational exchanges and reciprocity) also promotes effortful control (Kochanska et al., 2008; Li‐Grining, 2007), and verbal scaffolding techniques, such as labeling, build vocabulary and promote cognitive understanding of relations between objects and concepts, orienting children's attention and encouraging ongoing and extended engagement in activities. Collectively, these parent behaviors also promote child autonomy, provide emotional support, and enhance language development, inter‐related skills that are associated with effortful control and self‐regulation, more broadly (Baker, 2018; Bruce & Bell, 2022; Landry et al., 2002; Masek et al., 2021; Mermelshtine, 2017). Development of these skills prior to school lays the foundation for the self‐regulation required to learn effectively in school classrooms.

## Early parenting programs and support for self‐regulation development

Variations in the home environment partly explain associations between socioeconomic status (SES) and child self‐regulatory competencies. In the context of socioeconomic disadvantage, opportunities for cognitive stimulation in the family home may be limited. Fewer economic and time resources and greater stress can compromise parents' capacity to engage in language‐rich stimulating home activities with children. Findings from longitudinal observational studies support this contention (Lengua et al., 2007, 2014), with parenting practices accounting for some of the association between socioeconomic risk and children's effortful control. Research has also shown that a cognitively enriched home environment is one mechanism by which SES influences child inhibitory control (a component of effortful control). Specifically, higher parental education and income are associated with greater provision of cognitive stimulation, which in turn is related to higher child inhibitory control (Rosen et al., 2020). Parenting interventions that increase rich parent–child interactions and stimulating forms of play thus have the potential to mitigate the effects of early socioeconomic disadvantage on effortful control, with benefits for later learning. Moreover, meta‐analytic research has indicated that programs starting in infancy and toddlerhood tend to show larger effect sizes than programs delivered in the preschool years (Li et al., 2020), signifying that the early home environment may be a crucial and timely context for intervention delivery.

A range of early parenting programs delivered in infancy or toddlerhood have been designed to promote enriched home learning environments (e.g., Miller et al., 2023; Weisleder et al., 2019). However, few of these have been evaluated for their impact on either child effortful control or self‐regulation more broadly (Morawska et al., 2019). Three studies that have examined this have reported some or negligible gains in effortful control. The “Play and Learning Strategies” (PALS; Landry et al., 2008) intervention comprises 19 home‐based sessions for parents of 3‐ to 5‐year‐old children. It seeks to support children's language development by increasing responsive parenting behaviors including scaffolding, child‐led play and maintaining the child's focus and attention. The results from a randomized controlled trial with 623 families showed that parents participating in PALS reported improvements in children's effortful control at post‐intervention (6.5 months later) compared to parents in the ‘no PALS’ comparison condition (Landry et al., 2017; Merz et al., 2017).

*Family Check‐Up* is a long‐term, intensive program commencing when children are 2 years of age (Hentges et al., 2020). Annually, from ages 2 to 10.5 years, participating families are offered comprehensive family and child assessment, followed by provision of tailored parenting feedback and intervention. In a randomized controlled trial with 731 families, improvements in proactive parenting (structuring and scaffolding situations to meet the child's needs) at 3 years of age were indirectly related to greater effortful control at 5 years for those receiving this intervention (Chang et al., 2015). A follow‐up study using the same sample found that participation in *Family Check‐Up* was also associated with higher inhibitory control in middle childhood (8.5–10.5 years) (Hentges et al., 2020).

Finally, parents in the “Parents as Teachers (PAT)” program receive a minimum of 10 home visits per year for at least 2 years, commencing shortly after birth (Schaub et al., 2019). PAT aims to enhance parent knowledge of child development, promote early detection of developmental and health issues, and increase children's school readiness (Neuhauser, 2014). In a randomized controlled trial of 248 families, there was limited evidence of any gains in effortful control when children were 3 years old (Schaub et al., 2019). Significant improvements were seen for PAT families compared to the control condition, for one of the two measures of effortful control using non‐imputed data, with no significant findings for either measure when analyses were repeated with imputed data.

In summary, early parenting interventions that strengthen parents' capacity to provide an enriched home learning environment for their young children show some promise as an approach for enhancing child self‐regulation, including the effortful control skills that are important for successful classroom learning. However, evaluations that have assessed child self‐regulation or effortful control have been rare, and most have examined intensive, long‐duration individualized programs. Evaluations of these child outcomes at school age are lacking, and the effects of less‐intensive, group‐based programs are unknown. We address these gaps by examining school‐aged effortful control outcomes of a group‐based early childhood parenting program delivered during toddlerhood.

## The *smalltalk* early parenting intervention

The *smalltalk* program is a brief parenting intervention, developed in Australia, and fully integrated for delivery within existing early childhood services in the state of Victoria (Nicholson et al., 2016). It seeks to enhance the home learning environments of children aged 12 to 36 months from families with risk factors for poorer child outcomes (e.g., low income, single parent). The 10‐week program was conceptualized as a relatively ‘light‐touch’ approach embedded in existing facilitated playgroups to increase ease of reach, implementation, scale‐up and sustainability. *smalltalk* could be delivered in two formats: a lower intensity group‐only format (*smalltalk group‐only*); and a higher intensity format that supplemented the group program with six individual home coaching sessions (*smalltalk plus*).

In “standard” (i.e., non‐*smalltalk*) facilitated playgroups, parents and children meet at a community venue on a weekly basis. Facilitated playgroups in Australia consist of regular meetings with groups of parents and their children, providing opportunities for social interaction, play and development, led by a qualified facilitator who plans and guides the session (Commerford & Robinson, 2016). Trained facilitators structure session activities to guide parents through a range of developmentally appropriate play activities with their children (Commerford & Robinson, 2016).

In the 10‐week *smalltalk* playgroup sessions, facilitators introduce parents to core parenting strategies (one per week) to enhance their home learning environment. As summarized in Table 1, these include enriched home learning activities (e.g., “Supporting Children's Play”), high quality speech and descriptive language (e.g., “Listening and Talking More”), and responsive and attentive parent–child interactions that promote extended engagement in activities (e.g., “Following Your Child's Lead”) (Hackworth et al., 2017; Nicholson et al., 2016). Facilitators follow a written group session guide which describes how to discuss and model the parenting strategies in small groups, and how to provide opportunities for parents to practice and receive feedback. A number of resources are used including conversation cards, posters, and a take‐home DVD and practice worksheets. For *smalltalk plus*, parents receive six home coaching visits in addition to the group sessions. Home coaching sessions use the take‐home DVD as the focus for individualized support. The coach engages with parents as partners in their learning experience rather than experts. Coaches and parents jointly review each parenting strategy modeled on the DVD, work out how the parent could apply this, film the parent practicing and jointly review the footage.

**TABLE 1:** Short‐term outcome measures assessing the home learning environment and parent–child interactions.

*smalltalk*	Definition	Construct and measure			
		Home learning activities^a^	Parent verbal responsivity^a^	Parent descriptive language^b^	Extending child focus in interactions^b^
Parenting skills					
Learning through everyday routines	Routines that help children feel secure and provide a daily ‘infrastructure’ for parent–child interactions that promote learning and development	✓	✓		
Supporting children's play	Provision of developmentally appropriate play objects and activities essential for child development	✓			
Listening and talking more	Involves increasing exposure to language (both the frequency and variety of words) in a way that promotes ‘conversation’		✓	✓	
Using teachable moments	Involves capitalizing on everyday opportunities for learning. A teachable moment arises when a parent encourages a child to extend their knowledge or experience of something			✓	✓
Following the child's lead	Involves paying attention to and building on the child's interests				✓

*Note*: *smalltalk* parenting skills and definitions summarized from Nicholson et al. (2016).

^a^ Parent‐reported.

^b^ Observed behavior.

The effects of *smalltalk* playgroups were evaluated in a cluster‐randomized controlled trial with 1201 families randomized by location to one of three conditions: standard (usual care) playgroup, *smalltalk group‐only* or *smalltalk plus* (Hackworth et al., 2017). Evaluation data were collected post‐intervention and again at 5‐month follow‐up to assess short‐term outcomes listed in Table 1. At 5 months post‐intervention, parents in the *smalltalk group‐only* condition reported greater engagement in home learning activities and more verbal responsivity compared to the standard playgroup. Parents in the *smalltalk plus* group were observed to use more descriptive language and were more likely to engage in behaviors that maintained and extended their child's focus compared to the standard playgroup (Hackworth et al., 2017). In the current research, we examine whether these early changes are associated with later gains in child effortful control.

## The current study

Despite the potential for community‐delivered early childhood parenting programs to have wide reach and broad impact, long‐term follow‐ups are scarce. Few studies have assessed long‐term outcomes for these initiatives, and fewer still have examined distinct mechanisms of change (i.e., mediators). The current study addresses these research gaps. Specifically, the primary aim was to advance understanding of the mechanisms of change by examining potential mediating pathways between exposure to intervention versus usual care in an early childhood parenting intervention, and children's effortful control at school‐age, when children were approximately 7.5 years old.

**FIGURE 1:**
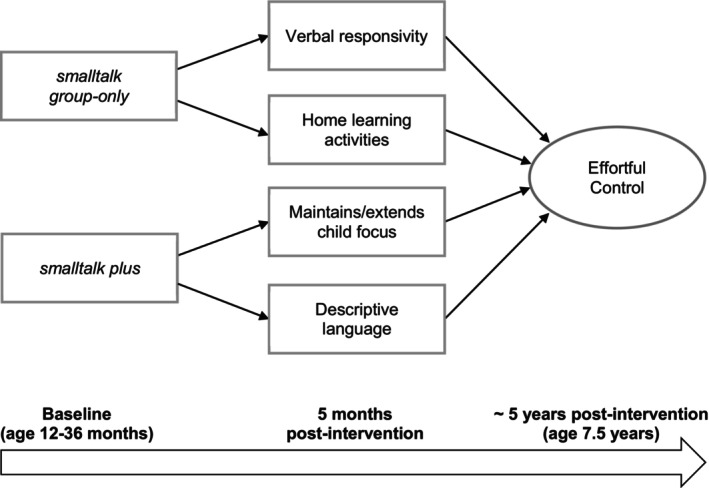
Hypothesized structural equation model of *smalltalk* and *smalltalk plus* influences on effortful control mediated through enriched parent–child interactions.

Mediators tested in the current study were four measures which demonstrated positive gains at follow‐up (Hackworth et al., 2017). Two were parent report measures (home learning activities, parent verbal responsivity) and two were direct observation measures (parent use of descriptive language, and maintaining and extending child's focus). For simplicity, we refer to these variables collectively as “enriched parent‐child interactions.” Our key outcome was effortful control. To capture this multi‐dimensional construct, we used a multi‐measure, multi‐informant approach with data collected from parents and teachers using three validated measures. It was hypothesized that more enriched parent–child interactions at 5‐month post‐intervention would explain (i.e., mediate) improvements in child effortful control assessed at 7.5 years, after adjusting for other demographic variables and indicators of social and economic disadvantage associated with parenting and effortful control (Figure 1).

A further aim of the study was to assess the effects that enriched parent–child interactions had on child effortful control over and above the effect of child vocabulary. Enriched parent–child interactions support both self‐regulation and language development in children. Child vocabulary is also associated with attentional and inhibitory subcomponents of effortful control (Razza et al., 2010; Weiland et al., 2014) and has been shown to partially explain the association between parenting behaviors and effortful control (Chang et al., 2015). Given the interconnected nature of children's self‐regulation and vocabulary, we assessed whether mediation effects persisted after adjusting for effects of child vocabulary.

## METHOD

### Participants and study design

Participants were 1201 parents of toddlers aged 12–36 months in 2010–2012 when originally recruited to the Early Home Learning Study (EHLS), a cluster randomized controlled trial of the *smalltalk* program (Nicholson et al., 2016). Parents were recruited through child and family service workers in 10 local government areas within the state of Victoria, Australia. Each area was divided into six geographically distinct locations, which were randomly assigned to deliver one of three study conditions: (i) standard playgroup (usual care), (ii) *smalltalk group‐only*, or (iii) *smalltalk plus*. Parents' home address was then used to determine their allocation to intervention condition.

Eligibility criteria included: living in a trial area; having at least one child aged 12–36 months; and evidence of at least one risk factor for social disadvantage including: low household income; receipt of government benefits (e.g., Health Care Card for low‐income families); single, socially isolated, or young parent (<25 years); or culturally and linguistically diverse background. Parents were not eligible if they were aged less than 18 years, did not speak sufficient English to participate, or were requiring more intensive support.

At baseline assessment, children had a mean age of 22.34 months (SD = 7.24), with a similar proportion of female (51.5%) and male (48.5%) children. Families reported one or more risk factors of social disadvantage, including low education (<grade 12, i.e., final year of high school; *n* = 139; 11.6%), low household income (*n* = 249; 21.6%), single parent household (*n* = 136; 11.3%), language other than English spoken at home (*n* = 395; 32.9%), young parent (≤25 years; *n* = 114; 9.5%) or unemployed household (*n* = 162; 13.5%). The state of Victoria in Australia is highly diverse in cultures and ethnicities. To reflect this, data were collected on language spoken at home and First Nations status, which were the most relevant sociocultural factors related to the program. The number of First Nations participants (<2%) was so small that this was not considered in analysis.

The school‐aged longitudinal follow‐up study, EHLS at School (Westrupp et al., 2018), was conducted between 2016 and 2018 and aimed to extend our early findings by assessing whether enriched parent–child interactions translated to longer‐term gains in children's outcomes in the early school years (child age 7.5 years). Families were invited to participate in EHLS at School approximately 5 years after their recruitment to the initial trial. To re‐engage participants, a study pack with EHLS at School Study engagement materials (study brochure, contact details form, magnet, and reply‐paid envelope) was sent to participants' last known home address requesting updated contact information. Participants were then contacted via phone and sent a children's book in appreciation. For those participants who did not respond, additional contact attempts were made via phone, email and/or text message. For participants still unable to be contacted, alternate contact details provided to us during EHLS (e.g., friends or family members) were used to track participants. Finally, Facebook tracing was attempted for 90 participants who remained uncontactable (Bennetts et al., 2021). Families were eligible for participation if they had not withdrawn from EHLS and had not declined future contact (*N* = 991). The study team became aware of one parent death prior to recruitment, reducing the number of eligible families to 990.

#### Retention

Of the 990 eligible families, 669 (67.6%) participated (standard playgroup: *n* = 200; *smalltalk group‐only*: *n* = 242, *smalltalk plus*: *n* = 227) and provided child outcomes data when children were approximately 7.5 years of age (*M*_age_ = 7.47, SD = 0.26). With written parental consent, children's teachers were also invited to participate and complete an online questionnaire. Families lost to attrition were more likely to come from lower income (29% vs. 16%) or unemployed households (19.2% vs. 9%), more socioeconomically disadvantaged neighborhoods (*M* = 4.0 vs. *M* = 5.0; higher values indicate more advantage), have parents who were younger (32.5 years vs. 34 years at baseline), had not completed grade 12 (i.e., final year of high school; 18.5% vs. 6.1%), were from single‐parent households (15.3% vs. 8.2%), and reported greater psychological distress (*M* = 4.1 vs. *M* = 3.6). All significant differences were at *p* < .001, except for psychological distress, which was significant at *p* < .05. See Table 2 for participant demographic characteristics.

**TABLE 2:** Sample characteristics of the EHLS and EHLS at School participants.

	Standard	*smalltalk*‐only	*smalltalk* plus	Overall
Baseline (EHLS)	(*n* = 351)^a^	(*n* = 410)^a^	(*n* = 440)^a^	(*n* = 1201)^a^
Parent age in years, *M* (SD)	33.25 (5.85)	33.52 (5.85)	33.21 (6.17)	33.32 (5.97)
Child age in months, *M* (SD)	21.74 (7.46)	22.37 (7.19)	22.79 (7.10)	22.34 (7.24)
Female parent, *n* (%)	327 (94.8)	388 (96.5)	417 (97.0)	1132 (96.2)
Female child, *n* (%)	170 (48.4)	211 (51.5)	238 (54.1)	619 (51.5)
Single parent household, *n* (%)	48 (13.7)	38 (9.3)	50 (11.4)	136 (11.3)
Low household income, *n* (%)	79 (23.8)	80 (20.4)	90 (21.1)	249 (21.6)
Unemployed household, *n* (%)	47 (13.4)	51 (12.4)	64 (14.5)	162 (13.5)
Parent low education (<grade 12), *n* (%)	42 (12.0)	48 (11.7)	49 (11.1)	139 (11.6)
Parent K6, *M* (SD)	3.70 (3.40)	3.54 (3.70)	4.16 (4.06)	3.81 (3.76)
SES rank, *M* (SD)	4.30 (2.57)	4.95 (2.54)	4.41 (2.79)	4.56 (2.66)
Non‐English speaking at home, *n* (%)	120 (34.3)	146 (35.6)	129 (29.3)	395 (32.9)
Follow‐up (EHLS at School)	(*n* = 200)^b^	(*n* = 242)^b^	(*n* = 227)^b^	(*n* = 669)^b^
Parent age in years, *M* (SD)	40.03 (5.24)	39.65 (5.14)	39.25 (5.79)	39.63 (5.40)
Child age in years, *M* (SD)	7.47 (0.30)	7.45 (0.23)	7.50 (0.26)	7.47 (0.26)
Parent K6, *M* (SD)	4.11 (3.39)	3.65 (3.00)	4.28 (3.12)	4.00 (3.17)
SES rank, *M* (SD)	5.09 (2.70)	5.71 (2.59)	5.11 (2.80)	5.31 (2.71)

*Note*: *N*s for measures vary at baseline and follow‐up.

Abbreviations: EHLS, Early Home Learning Study; K6, Kessler 6 (parent psychological distress); SES rank, neighborhood socioeconomic status according to Socio‐Economic Indexes for Areas Index of Relative Socioeconomic Advantage and Disadvantage deciles.

^a^ Refers to sample size at baseline.

^b^ Refers to sample size at EHLS at School follow‐up.

### Measures

#### Mediators: Enriched parent–child interactions (5 months post‐intervention)

##### Home learning activities

The 5‐item parent‐report scale from the Longitudinal Study of Australian Children was used to assess the frequency with which parents engaged in home learning activities with their child (e.g., “read books to your child”). The scale is a modified version of the Early Childhood Longitudinal Study, Kindergarten Cohort measure (National Center for Education Statistics, 2000). Items were scored on a 4‐point scale ranging from 1 = ‘not at all’ to 4 = ‘everyday’. Higher scores indicate more frequent engagement in home learning activities. Acceptable reliability and theoretically consistent associations with children's emotional‐behavioral resilience have been documented in previous research (Giallo et al., 2018; Nicholson et al., 2008). Cronbach's *α* was .62 for the current sample.

##### Parent verbal responsivity

The 4‐item parent‐report subscale of the StimQ‐T (Dreyer et al., 1996) was used to assess the frequency of parents' verbally responsive behaviors (e.g., “I talk about the day while my child is eating”). Items were scored on a 4‐point scale ranging from 1 = ‘not at all’ to 4 = ‘everyday’. Higher scores indicate more frequent verbal responsivity. Construct and convergent validity with the Infant‐Toddler version of the Home Observation Measure of the Environment have been demonstrated for the parent verbal responsivity subscale (Dreyer et al., 1996) Cronbach's *α* was .52 for the current sample.

##### Parent–child interaction

The Indicator of Parent–Child Interaction (IPCI; Baggett et al., 2010) was used as a direct in‐home observational measure of parent–child interactions. Parent–child dyads were asked to engage in four common early childhood activities, including free play, looking at books, a distraction task, and getting dressed, resulting in approximately 8–10 min of video footage. Videos were coded for 14 domains by two independent postgraduate research assistants at the University of Kansas, under the supervision of the research scientist who developed the IPCI [KB]. Coders had completed certification training prior to coding, which involved a written test and engaging in half‐day trainings with sample assessments. Eighty percent agreement with a gold‐standard rater is required to achieve certification. Reliability was reviewed in weekly meetings with 20% of videos within each weekly batch of coding assignments randomly selected and assigned to an independent coder for the purpose of calculating inter‐rater agreement. If individual coder agreement fell below the 80% criterion, areas of disagreement were discussed, and additional coding practice was assigned until criterion was achieved and a new study coding batch was assigned. Overall study inter‐rater agreement exceeded 80% among 20% of independently coded videos, consistent with prior research (Baggett & Carta, 2006; Harknett et al., 2022; Moore, 2018). Due to time and budget constraints, a random sample of approximately 20% (participants with both baseline and 5‐month follow‐up data) was coded. Of this sample, some video data could not be coded due to data quality issues, reducing the final sample slightly (final *N* = 151). Using updated occurrence coding (see Harknett et al., 2022; Moore, 2018), videos were coded for frequency of behaviors in each domain. Specifically, parent behaviors were coded in 30‐s intervals as present or absent (1 or 0, respectively). As there was some variability in the length of videos and the number of intervals able to be coded, the summary score was the number of intervals where the behavior was present divided by the total number of intervals coded, expressed as a percentage. Two of the 14 IPCI domains are reported here: (1) “*Parent use of descriptive language*” and (2) “*Parent maintains and extends child's focus*”. These scales assess the extent to which the parent (1) uses rich descriptive language to label objects and people in connection with actions and adjectives (e.g., child points to a dog, and parent responds “Yes, that's a doggie, you see the brown doggie”), and (2) engages in behaviors that maintain and extend the child's interest in an activity, leading to continued engagement (e.g., child is engaging in pretend play with farm animals and picks up an animal. Parent makes an animal noise and suggests feeding the animal). Adequate psychometric properties have been demonstrated for the IPCI (Baggett & Carta, 2006), including theoretically expected associations between the two domains reported in this study and other observational scales of parent–child interaction (Harknett et al., 2022).

#### Effortful control at school age (5 years post‐intervention)

A multi‐measure, multi‐informant approach was used to capture three elements of children's effortful control (attentional control, learning‐related regulatory behaviors, and inhibitory control) across two key settings (home and school). A latent variable for effortful control was created using five measures: summed total of three items from the Strengths and Difficulties Questionnaire (SDQ; Goodman, 1997), (i) parent‐ and (ii) teacher‐report; the total score of the Approaches to Learning Scale (National Center for Education Statistics, 2000), (iii) parent‐ and (iv) teacher‐report; and (v) one subscale from the Temperament in Middle Childhood Questionnaire, parent‐report only (TMCQ; Simonds & Rothbart, 2004).

The SDQ is a multi‐dimensional instrument for assessing children's social, emotional, and behavioral difficulties across different contexts. Three items representing attentional control from the 5‐item hyperactivity/inattention subscale of the SDQ (parent‐ and teacher‐report) were selected to capture attentional components of effortful control and summed to generate a total score (items: “easily distracted, concentration wanders (R)”, “thinks things out before acting”, and “good attention span, sees chores or homework through to the end”). Items are rated on a 3‐point scale of 0 = ‘not true’, 1 = ‘somewhat true’, and 2 = ‘certainly true’. Higher scores indicate a greater degree of attentional control. Differentiation of the hyperactivity component from the inattention component has been indicated by item response theory analysis of the SDQ (Keller & Langmeyer, 2019) and acceptable internal consistency for the three items has been reported in previous research (von Salisch et al., 2017). Adequate reliability and validity for the SDQ have been established for both the parent and teacher report (Stone et al., 2010). Cronbach's *α* for parent‐ and teacher‐report were .69 and .84 respectively for the current sample.

The Approaches to Learning scale was selected to assess components of children's effortful control within the context of learning‐based activities particularly relevant to school‐aged children. In early to middle childhood, there is a notable increase in time dedicated to learning activities, and how children approach these activities reflects their self‐regulatory capabilities (Li‐Grining et al., 2010). The 6‐item Approaches to Learning scale (parent‐ and teacher‐report) assesses children's learning‐related regulatory behaviors, including persistence, concentration, attention, and responsibility (e.g., “persists in completing tasks”). Items are rated on a 4‐point scale from 1 = ‘never’ to 4 = ‘very often’. Higher scores indicate a greater degree of learning‐related regulatory behaviors. Good internal consistency and predictive validity have been demonstrated for this measure (Berthelsen et al., 2017). Cronbach's *α* for parent‐ and teacher‐report were .80 and .91 respectively for the current sample.

Finally, the *inhibitory control* subscale of the TMCQ (parent‐report only) was used to measure inhibitory control aspects of effortful control. This assesses children's ability to suppress inappropriate prepotent responses in favor of more adaptive goal‐consistent responses. This scale comprises eight questions related to behavioral inhibition (e.g., “can stop him/herself from doing things too quickly”), rated on a 5‐point Likert‐type scale from 1 = ‘almost always untrue’ to 5 = ‘almost always true’. Higher ratings indicate a greater degree of inhibitory control. Acceptable internal consistency has previously been established for this subscale (Kotelnikova et al., 2017). The subscale is also correlated in theoretically expected ways with diagnostic and parent‐report measures of inattention and hyperactivity (Herzhoff et al., 2013) as well as a physiological measure of dysregulation (Kil et al., 2023). Cronbach's *α* was .69 for the current sample.

#### Child vocabulary at school age (5 years post‐intervention)

As a measure of receptive vocabulary at child age 7.5 years, the National Institutes of Health (NIH) Toolbox Picture Vocabulary Test (Weintraub et al., 2013) was administered via iPad in a computerized adaptive format. Children are presented with four pictures on an iPad screen and an audio recording of a word. Children are then instructed to select the image that best represents the word. There are a maximum of 25 items, with the difficulty of presented words dependent on the response given to the prior item. Raw scores are converted to standard scores adjusted for age with a mean of 100 and a standard deviation of 15. The measure was modified with permission from the NIH to use an Australian accent for audio recording of items.

#### Covariates

The following child‐, family‐, household‐, and neighborhood‐level demographic variables collected at baseline were included in the analysis: child age and sex (coded as 0 = male, 1 = female), language other than English spoken at home (0 = no, 1 = yes), single‐parent household (0 = married/living with partner, 1 = single, divorced, or separated), parent education (0 = grade 12 education or higher, 1 = less than grade 12 education), parent psychological distress, assessed using the K6 (Kessler et al., 2002), neighborhood‐level SES based on participant postcode, assessed using the area‐based Socio‐Economic Indexes for Areas (SEIFA) Index of Relative Socioeconomic Advantage and Disadvantage (Australian Bureau of Statistics, 2013) and ranked in deciles (10 = greatest advantage, 1 = greatest disadvantage), and baseline score of child temperament assessed using the 4‐item Mood and Behavior subscale of the NEILS Scales of Developmental Competency (higher scores indicate more difficulties with mood and behavior; Hebbeler et al., 2007). Finally, child‐ and family‐level covariates at the school‐aged assessment included parent psychological distress (K6) and neighborhood‐level SES (SEIFA; Australian Bureau of Statistics, 2018). These variables were selected to account for potential differences in effortful control and parenting by social and economic factors as indicated by prior research, i.e., single‐parent household, parent education, language other than English spoken at home, neighborhood SES, and parent psychological distress (e.g., Li‐Grining, 2007; Merz et al., 2014). Child age and sex were also adjusted for because gender differences have been observed for children's effortful control (Else‐Quest et al., 2006). Finally, to account for temperamental antecedents of effortful control, a baseline measure of temperament was included in the models.

### Statistical analyses

Analyses were conducted in R version 4.1.0 (R Core Team, 2021) using the *lavaan* package (Rosseel, 2012). A preliminary structural equation model (SEM) was first conducted to confirm that the three attention items of the SDQ were distinct from the two hyperactivity items from the same measure and therefore appropriate for use in the model independently, i.e., without the hyperactivity component of the subscale. A measurement model was then specified to evaluate the latent construct of effortful control (represented by inhibitory control, attentional control, and approaches to learning).

We subsequently analyzed the indirect effects, i.e., mediation effects, of the *smalltalk* intervention on children's effortful control via each of the four separate mediators of enriched parent–child interactions, resulting in four separate models using SEM. A single multiple mediator model was specified thereafter to evaluate the hypothesized joint mediation effects of the four mediators combined into a single model. This allowed us to determine if the set of four parent–child interaction variables jointly mediated the effect of the intervention on children's effortful control, and then to assess the extent to which each specific parent–child interaction variable acted as a mediator, accounting for the other mediators in the model. To determine whether mediation effects persisted after adjusting for child vocabulary, a final model was specified which included all variables from the joint mediation model and child vocabulary as a covariate. Cluster‐robust sandwich estimators were used to account for the nested structure of the data (i.e., cluster randomization by geographical location).

In all models, the latent dependent variable of child effortful control at 5 years post‐intervention was regressed onto the mediating variables (home learning activities, parent verbal responsivity, parent use of descriptive language, maintaining and extending child's focus). Mediating variables were regressed onto the independent variable of treatment condition, which was dummy coded for *smalltalk group‐only* and *smalltalk plus*, with the standard condition treated as the reference category. Models controlled for the following child and family covariates at baseline: child age, sex, and temperament, parent psychological distress, language other than English spoken at home, single‐parent household, low income, household unemployment, parent age, and neighborhood‐level SES. Most of these variables were also associated with missingness from baseline to school‐age follow‐up. To reduce multicollinearity, and as the more proximal indicators of these constructs, parent psychological distress and neighborhood‐level SES at school‐age follow‐up were adjusted for in paths predicting effortful control, rather than their baseline counterparts. Finally, we controlled for baseline scores on the mediator variables (home learning activities, parent verbal responsivity, parent use of descriptive language, and maintaining and extending child's focus). Child age, sex, parent psychological distress, language other than English spoken at home, single‐parent household, and neighborhood‐level SES were entered to predict all mediators in the model. Child sex and baseline temperament, parent psychological distress, neighborhood‐level SES, and language other than English spoken at home were entered to predict the effortful control outcome.

All exogenous variables were allowed to covary. To account for common method variance, additional residual covariances included in the model were between: parent‐reported parent verbal responsivity and home learning activities; observational measures of descriptive language and maintaining/extending child's focus; and teacher‐reported indicators of effortful control. Observational variables were re‐scaled from percentages to a 0 to 10 scale to facilitate estimation of models (Little, 2013). Finally, modification indices indicated the following improvements to the multiple mediator model: home activities at baseline predicted parental verbal responsivity at follow‐up and parent verbal responsivity at baseline predicted home activities at follow‐up. Indirect effects were assessed using the product of coefficients approach and Monte Carlo confidence intervals. The Monte Carlo method uses simulation draws to produce the sampling distribution of indirect effects. It performs comparably and is less computationally intensive than other approaches to constructing confidence intervals (MacKinnon et al., 2007; Preacher & Selig, 2012). Indirect effects were interpreted as significant if the 95% confidence interval did not cross zero. Standardized estimates are reported.

Number of observations with available data for each variable are presented in Table 3 alongside bivariate correlations (missing data on variables ranged from 87.4% to 21.7%). Only a random subsample of data was coded for the IPCI, resulting in smaller numbers and a higher proportion of missing data for the two observational outcome measures. Models were conducted with full estimation maximum likelihood, which accounted for missing data (Enders, 2010). Robust fit indices were requested to account for any non‐normality of the data. Model fit was assessed using the Chi square test (*χ* ^2^) in conjunction with other model fit indices because the Chi square test can be overly sensitive in large samples (Kline, 2015). Additional model fit indices were the Tucker–Lewis index (TLI), the comparative fit index (CFI), and root mean square error of approximation (RMSEA). The model was deemed to have adequate fit if the RMSEA was close to or less than .06, and the TLI and CFI were at least .90. A TLI and CFI of .95 or higher indicated a very good fit (Byrne, 2012; Hu & Bentler, 1999). Though analyses were not explicitly preregistered, our choice of variables was guided by theory and prior research, and our analyses should therefore be considered predominantly confirmatory.

**TABLE 3:** Correlations and summary statistics for study variables.

	1	2	3	4	5	6	7	8	9	10	11	12	13	14	15	16	17	18	19	20	21	22
Baseline covariates																						
1. Parent age (in years)	‐																					
2. Child age (in months)	.09**	‐																				
3. Child gender	−.03	−.01	‐																			
4. Language other than English	−.01	.03	−.03	‐																		
5. Low household income	−.11***	.01	−.02	.08**	‐																	
6. Unemployed household	−.03	.01	.01	.00	.60***	‐																
7. Single parent household	−.03	.00	.02	−.10***	.55***	.63***	‐															
8. Parent education (<grade 12)	−.12***	.03	.04	−.05	.30***	.38***	.27***	‐														
9. K6	−.08**	.05	.05	.13***	.18***	.12***	.12***	.06*	‐													
10. SES rank	.14***	−.12***	−.03	−.21***	−.20***	−.15***	−.10***	−.12***	−.11***	‐												
11. Child temperament	−.03	.22***	.10***	.05	.05	.05	.03	.05	.23***	−.04	‐											
Follow‐up covariates																						
12. K6	−.06	.00	.00	.08*	.16***	.11**	.12**	.04	.41***	−.09*	0.19***	‐										
13. SES rank	.12**	−.12**	.01	−.12**	−.13***	−.14***	−.09*	−.11**	−.07	.68***	−0.01	−.05	‐									
Enriched parent–child interactions																						
14. Home activities	.00	−.10**	−.05	−.19***	−.06	−.02	−.01	−.04	−.14***	.15***	−0.12***	−.11**	.07	‐								
15. Verbal responsivity	−.05	−.22***	−.05	−.03	.03	−.01	−.03	.07*	−.05	.01	−0.15***	−.10*	−.01	.52***	‐							
16. Descriptive language	.10	.10	−.06	−.32***	−.12	−.06	−.09	.05	−.11	.07	.02	−.04	.03	.19*	.08	‐						
17. Extends child's focus	.12	.26**	−.02	−.23**	−.08	−.08	−.09	.00	−.08	−.08	.06	−.06	−.08	.20*	.04	.47***	‐					
Effortful control indicators																						
18. Inhibitory control (P)	−.01	.02	−.18***	−.05	−.02	−.03	−.03	−.10**	−.16***	.04	−.17***	−.21***	.02	.11**	.07	−.01	.08	‐				
19. Approaches to learning (P)	.02	.00	−.19***	.02	−.02	−.04	−.02	−.13***	−.13**	.05	−.13***	−.16***	.07	.19***	.13**	−.02	.20*	.43***	‐			
20. Approaches to learning (T)	.05	.02	−.35***	.00	.00	−.04	−.01	−.12**	−.03	.05	−.06	−.02	.04	.05	.02	.06	−.02	.35***	.39***	‐		
21. Attentional control (P)	.05	.06	−.20***	.11**	.04	.02	−.02	−.10*	−.13**	.01	−.16***	−.21***	.02	.05	.06	.01	.17	.57***	.61***	.47***	‐	
22. Attentional control (T)	.08	.04	−.33***	−.01	−.02	−.03	.01	−.09*	−.03	.05	−.06	−.04	.00	.04	−.03	−.02	.14	.34***	.41***	.81***	.48***	‐
*N*	1199	1200	1201	1200	1151	1200	1200	1200	1195	1201	1196	647	665	938	940	151	151	653	651	470	651	463
*M*	33.32	22.34	0.52	0.33	0.22	0.14	0.11	0.12	3.81	4.56	6.30	4.00	5.64	17.62	12.90	66.32	26.85	3.46	3.21	3.30	3.53	3.89
SD	5.97	7.24	0.50	0.47	0.41	0.34	0.32	0.32	3.76	2.66	1.70	3.17	2.66	2.35	2.29	24.21	25.21	0.62	0.53	0.68	1.55	1.89

Abbreviations: K6, parent psychological distress; *M*, mean; *N*, number of observations; P, parent‐report; SD, standard deviation; SES rank, socioeconomic status according to Socio‐Economic Indexes for Areas Index of Relative Socioeconomic Advantage and Disadvantage deciles; T, teacher‐report.

* *p* < .05;

** *p* < .01;

*** *p* < .001.

## RESULTS

**FIGURE 2:**
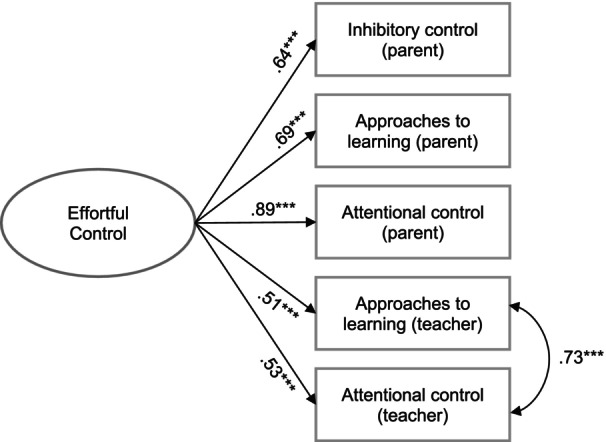
Standardized coefficients of measurement model for latent variable of effortful control. ^***^ *p* < .001

Demographic characteristics of all participants are presented in Table 2, at baseline (EHLS) and five‐year follow‐up (EHLS at School). Correlations between study variables are presented in Table 3. Preliminary analyses confirmed that the three SDQ attention items were distinct from the two hyperactivity items (Parent‐report: *χ* ^2^(3) = 0.56, *p* = .91; CFI = 1; TLI = 1, RMSEA = 0; Teacher‐report: *χ* ^2^(3) = 3.32, *p* = .35; CFI = 1; TLI = 1, RMSEA = .02). The results from the measurement model for the latent factor of effortful control also indicated very good model fit (*χ* ^2^(4) = 2.22, *p* = .70; CFI = 1; TLI = 1, RMSEA = 0). See Figure 2 for standardized parameter estimates of latent variable indicators.

### Single mediator models

All single mediator models fit the data well, with similar fit statistics across models. See Table 4 for parameter estimates and fit statistics for all single mediator models. In the single mediation model for home learning activities, only the path between home learning activities and effortful control was significant (*β* = .09, *p* < .05), with no significant mediation effects. In the single mediation model for parent verbal responsivity, the *smalltalk group‐only* program was associated with higher parental verbal responsivity at 5‐month post‐intervention (*β* = .07, *p* < .05), but no other paths were significant. There was also no mediation effect observed. In the single mediation model for parent descriptive language use, the *smalltalk plus* program was associated with more descriptive language use at 5‐month post‐intervention (*β* = .20, *p* < .01), but no other paths were significant, and no mediation effect was observed. Finally, in the single mediation model for ‘extends and maintains child focus,’ a significant association was observed between *smalltalk plus* and parents' capacity to maintain and extend their children's focus (*β* = .25, *p* < .001). A significant association was also observed between this variable and children's effortful control at age 7.5 years (*β* = .25, *p* < .001). The mediation effect was significant, with parents' capacity to maintain and extend their children's focus mediating the effect of the *smalltalk plus* program on children's later effortful control (indirect effect: *β* = .06, *p* < .01, Monte Carlo CI [0.02, 0.11]).

**TABLE 4:** Direct and indirect paths from single mediator models.

Paths	*B*	SE	*β*	Model fit			
				CFI	TLI	RMSEA	*χ* ^2^ (df)
Home learning activities				.96	.95	.026	150.27 (81)***
*smalltalk group‐only* → Home learning activities	.28	.17	.06				
*smalltalk plus* → Home learning activities	.22	.16	.05				
Home learning activities → EC (7.5 years)	.02*	.01	.09*				
*smalltalk group‐only* → Home learning activities → EC (7.5 years)	.00	.00	.01				
*smalltalk plus* → Home learning activities → EC (7.5 years)	.00	.00	.00				
Parent verbal responsivity				.97	.95	.025	141.87 (81)***
*smalltalk group‐only* → Verbal responsivity	.32*	.16	.07*				
*smalltalk plus* → Verbal responsivity	.09	.14	.02				
Verbal responsivity → EC (7.5 years)	.01	.01	.05				
*smalltalk group‐only* → Verbal responsivity → EC (7.5 years)	.00	.00	.00				
*smalltalk plus* → Verbal responsivity → EC (7.5 years)	.00	.00	.00				
Parent descriptive language				.96	.95	.023	136.58 (81)***
*smalltalk group‐only* → Descriptive language	.33	.37	.06				
*smalltalk plus* → Descriptive language	1.01**	.35	.20**				
Descriptive language → EC (7.5 years)	.02	.02	.13				
*smalltalk group‐only* → Descriptive language → EC (7.5 years)	.01	.01	.01				
*smalltalk plus* → Descriptive language → EC (7.5 years)	.02	.02	.03				
Maintains and extends child's focus				.97	.95	.023	133.68 (81)***
*smalltalk group‐only* → Maintains/extends focus	.21	.27	.04				
*smalltalk plus* → Maintains/extends focus	1.32***	.33	.25***				
Maintains/extends focus → EC (7.5 years)	.04***	.01	.25***				
*smalltalk group‐only* → Maintains/extends focus → EC (7.5 years)	.01	.01	.01				
*smalltalk plus* → Maintains/extends focus → EC (7.5 years)	.05**	.02	.06**				

*Note*: Models included child and family covariates and baseline levels of mediators.

Abbreviations: *B*, unstandardized coefficient; CFI, comparative fit index; EC, effortful control; RMSEA, root mean square error of approximation; SE, standard error; TLI, Tucker‐Lewis index; *β*, standardized coefficient.

* *p* < .05;

** *p* < .01;

*** *p* < .001.

### Multiple mediator model

**FIGURE 3:**
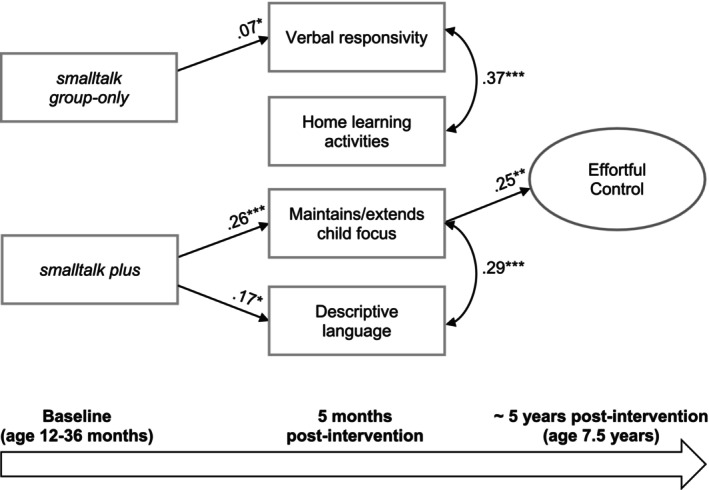
Standardized coefficients of observed structural equation model, showing results (*p* < .05). Intervention effects for two intervention conditions (*smalltalk group‐only* and *smalltalk plus*) are compared against the standard (usual care) condition. Single‐headed straight lines represent standardized coefficients where *p* < .05. Paths where *p* > .05 are not reported. The analysis is adjusted for baseline scores of mediators, child age and sex, parent psychological distress, language other than English spoken at home, single parenthood, low household income, household unemployment, parent age, parent education, neighborhood‐level socioeconomic status (SES), child temperament, and school‐aged assessment of parent psychological distress and neighborhood‐level SES; *p < .05. **p < .01 ***p < .001.

The overall path model fit the data well (*χ* ^2^(131) = 202.29, *p* < .001; CFI = .97, TLI = .96, RMSEA = .021). Figure 3 shows significant standardized coefficients from the SEM model. The *smalltalk group‐only* condition was associated with higher levels of parent verbal responsivity 5 months post‐intervention, after adjusting for baseline levels of verbal responsivity, home activities, and other sociodemographic covariates. There was also an association between the *smalltalk plus* condition and parents' increased capacity to maintain and extend their children's focus in activities at 5‐month post‐intervention, and an association between *smalltalk plus* and parents' use of descriptive language at 5‐month post‐intervention, after adjusting for other covariates. Finally, parents' capacity to maintain and extend their children's focus in activities was positively associated with effortful control at school age (7.5 years), after adjusting for other mediators.

We did not find a significant joint mediation effect for the *smalltalk group‐only* condition with the combined set of mediators (total indirect effect: *β* = .01, *p* = .40, Monte Carlo CI [−0.02, 0.05]), but there was a joint mediation effect for the *smalltalk plus* intervention (total indirect effect: *β* = .05, *p* = .02, Monte Carlo CI [0.01, 0.11]). Examination of the Monte Carlo confidence intervals indicated only one significant unique indirect effect. Specifically, we found an indirect effect from the *smalltalk plus* condition to children's effortful control at school age, mediated by higher levels of parenting practices that involved maintaining and extending children's focus in activities (indirect effect: *β* = .07, *p* = .01, Monte Carlo CI [0.02, 0.12]). This effect was similar to what was found in the single mediator model. The results from the multiple mediator model indicate unique indirect effects, after accounting for other mediators. We also tested direct paths between the intervention conditions (*smalltalk group‐only* and *smalltalk plus*) and effortful control, accounting for the mediator variables, but neither path was statistically significant, and model fit was marginally, although not significantly, worse.

### Adjustment for child vocabulary in multiple mediator model

The inclusion of child vocabulary in the model did not substantively alter results. Child vocabulary was also not uniquely related to child effortful control once other covariates had been accounted for. Fit statistics for the model were similar: *χ* ^2^(139) = 215.93, *p* < .001; CFI = .97, TLI = .95, RMSEA = .021.

## DISCUSSION

The aim of this study was to examine the mechanisms of change (mediators) by which an early childhood parenting intervention delivered when children were aged 12–36 months impacted children's effortful control at age 7.5 years. Potential mechanisms of change were investigated by testing whether higher child effortful control at school‐age was explained by earlier changes in parenting and the early home learning environment, across the original two intervention groups of the EHLS, compared to the standard (usual care) condition. We examined potential mediating pathways between exposure to *smalltalk* and children's effortful control first through single mediator models and then by assessing the combined (joint) mediation effects of all four enriched parent–child interaction variables, as well as unique indirect effects, that is the mediation effect of each individual parent–child interaction variable adjusting for the effects of the other parent–child interaction variables. A joint mediation effect of the four enriched parent–child interaction variables was observed between *smalltalk plus* and effortful control at age 7.5 years, and we found one unique indirect effect. Specifically, compared to the standard condition and after accounting for other intervention outcomes, *smalltalk plus* was associated with increases in parents' capacity for ‘maintaining and extending the child's focus’, which then mediated the effect of the intervention on children's school‐aged effortful control. Similar patterns were not found for the other three enriched parent–child interaction variables: parent verbal responsivity, home learning activities, and descriptive language use. This unique indirect effect suggested that the joint mediation effect of the set of enriched parent–child interaction variables on children's effortful control was largely due to improvements in parents' capacity to maintain and extend their children's focus. For the *smalltalk group‐only* intervention compared to the standard condition, no indirect effects were found for any of the four parent–child interaction variables. These findings were also consistent with results from the single mediator models.

Our study findings extend early parenting intervention research by providing a long‐term follow‐up of an intervention study in a large population‐based sample, and a direct examination of an important child outcome seldom examined in these studies (i.e., child effortful control). Despite the importance of self‐regulation for later academic outcomes, health, and psychological wellbeing, few studies have evaluated the impact of early childhood parenting interventions on self‐regulation in childhood (see Morawska et al., 2019 for a review). Likewise, randomized controlled trials of early parenting interventions with long‐term follow‐ups remain scarce, with few studies conducting follow‐ups past 12 months (see e.g., Rayce et al., 2017).

Our study builds on the findings from other programs such as “PALS” (Landry et al., 2008) and “Family Check‐Up” (Chang et al., 2015; Hentges et al., 2020) in a number of important ways. First, our data support the findings from these studies that early childhood interventions which successfully promote an enriched home environment can bring important benefits in terms of effortful control. Our findings demonstrated that all four enriched parent–child interaction variables jointly mediated the effects of *smalltalk plus* on effortful control, with examination of unique effects indicating that this was largely due to improvements in parents' capacity to promote children's extended engagement in activities and play. However, we also provide new evidence that demonstrates that these gains are possible with an intervention approach that is less intensive than either PALS or Family Check‐Up. The novel combination of a playgroup‐based program combined with home coaching brings together a range of effective intervention elements. In the *smalltalk* playgroup, active teaching (e.g., discussion, modeling, practice, and feedback) is provided to multiple parent–child dyads simultaneously, creating opportunities for peer modeling and support. However, our findings suggest that this may be insufficient on its own to effect change. The six home‐based coaching sessions provided in the *smalltalk plus* condition allowed parents to consolidate group learnings, and to engage in one‐on‐one practice and feedback, with a focus on how parents could translate the program strategies to their own child and personal circumstances. Our study shows that the addition of this home coaching component may be necessary for enhancing children's later effortful control.

Our study also demonstrates an enduring effect beyond those previously found with long‐term, intensive, individual early parenting programs. For PALS, gains in effortful control were reported for 3‐ to 5‐year‐olds at post‐intervention (Landry et al., 2017). PAT reported weak effects on effortful control for 3‐year‐olds at post‐intervention. For Family Check‐Up, the follow‐up evaluation when children were aged 8.5 to 10.5 showed gains in inhibitory control, while the attentional components of effortful control that are important for children's development and learning were not assessed (Kim‐Spoon et al., 2019; Valiente et al., 2011). Moreover, inhibitory control was assessed by caregiver‐report only, and may not have reflected children's performance in a school context. Comprehensive assessment of effortful control is particularly important in middle childhood, as the environmental demands of the classroom increase, and children are exposed to more varied and complex social interactions. Sustained attention to lessons, resisting distractions, and engaging in appropriate behavior with peers and teachers are facilitated by higher levels of effortful control and can lead to better school adjustment (Eisenberg et al., 2010; Valiente et al., 2008). Middle childhood may also represent a critical developmental window for assessing the effects of parenting programs on effortful control. Effortful control stabilizes from pre‐adolescence onwards (Tiberio et al., 2016) and the effects of early parenting may become less salient.

Finally, our study significantly builds new knowledge by providing a rare examination of mechanisms of intervention change over long‐term follow‐up. Four potential mediators were examined. Parents' ability to extend and maintain their child's focus was identified as the key variable. Although our findings broadly support the importance of parental involvement in children's learning and play during toddlerhood for later self‐regulation, they also suggest that verbal interactions and dyadic engagement in activities alone may be insufficient to promote effortful control in later years. Rather, it seems necessary for parenting to include an element of scaffolding—parenting practices that are child‐led and promote continued or extended engagement in an activity. Such behaviors are proposed to support autonomy and encourage sustained attention for task completion and are foundational for supporting child self‐regulatory competencies (Carlson, 2003; Fay‐Stammbach et al., 2014; Matte‐Gagné & Bernier, 2011; Neale & Whitebread, 2019).

In contrast to the *smalltalk plus* condition, mediation effects were not observed for the *smalltalk group‐only* condition. This suggests that the guided home‐based practice component as an addition to the group‐based programs is needed to promote the advanced parenting skills that have persistent effects for children's development. Specifically, in addition to the group‐based playgroups, parents in the *smalltalk plus* condition were offered six bi‐weekly home coaching sessions, in which parents watched videos of parenting skills and discussed with a home coach ways to practice and apply these skills in day‐to‐day parent–child interactions. The home coaching sessions afforded parents the opportunity to gain greater understanding, practice, and generalization to the home setting, which may have been particularly important for promoting gains in higher‐level parenting skills such as the capacity to extend children's interest in activities. These sessions also included video feedback (i.e., guided viewing of parent–child interactions with feedback), which is believed to be an effective tool for encouraging self‐reflection and adaptation of parenting practices (Fukkink, 2008). In sum, our findings suggest that for a light‐touch, group‐based program to result in longer‐term gains in children's self‐regulation the use of intervention strategies that offer more individualized and guided feedback may be required to promote mastery of more advanced parenting skills. Importantly, our research shows that this can be achieved from just 16 sessions (10 group sessions and 6 at home) delivered over 10 weeks.

### Strengths and limitations

Our study has several notable strengths, including the rigorous large‐scale study design and good retention 5 years post intervention. We used a range of strategies to overcome the limitations inherent to using informant data for assessing child development. For example, we used comprehensive and diverse data collection methods, including parent‐reported, teacher‐reported, and direct observational data. Although our sample of direct observational data was smaller than our parent‐ and teacher‐reported data due to coding time and cost constraints, it was a relatively large sample for data of this nature and was sufficient for the detection of significant associations.

Our outcome measure of effortful control also relied on indicators from both parent‐ and teacher‐report. This enabled us to capture different perspectives on the child's ability to self‐regulate across two key childhood contexts—the classroom setting and home environment. Although parents are better positioned than teachers to comment on their children's behavior across multiple timepoints and contexts, there is also the potential for parents' responses to be influenced by conscious or unconscious biases, such as social desirability, mental health conditions, language proficiency, or varied understandings of the questions being asked (Bennetts et al., 2016). Our use of teacher‐report in conjunction with parent‐report would to some extent have mitigated effects of parents' socially desirable responding, since teacher‐reports are characteristically made in the context of a broad understanding of children's development and in comparison with their classroom peers. Although we note that similar to parent‐report, teacher‐report may also be subject to some reporting bias.

To further circumvent some of these measurement issues, our analyses adjusted for factors such as parental mental health and speaking a language other than English at home. An alternative approach used by some researchers is the direct assessment of effortful control using performance‐based measures. This was beyond the scope of the present study, and direct assessments also have limitations. They may fail to capture children's naturalistic performance, and when assessed at a single timepoint, can be overly influenced by contextual factors, such as child sickness or tiredness (Campbell et al., 2016).

An additional limitation pertains to two of the parenting measures used in this study, each consisting of 4 to 5 items, with levels of internal consistency that would be considered low by the standards of the field. These measures were chosen to minimize participant burden, however the limited number of items can reduce internal consistency and may only capture some aspects of a broad spectrum of early learning activities and interactions. We cannot therefore discount the possibility that additional indirect effects may have been observed with different measures. Similarly, there may be cultural differences in the home environment that are less precisely captured when using a brief index of activities. Although research using similar item indices provides some support for cross‐cultural relevance (Aminipour et al., 2020) and use in culturally diverse populations (e.g., Malhi et al., 2018; Roby et al., 2021), these are issues that nonetheless require consideration for future research. A limitation related to the observational measure of parent–child interaction should also be noted. Specifically, the number of intervals available for coding varied for some participants, which may have afforded them fewer opportunities to demonstrate parenting behaviors of interest.

Finally, we note a limitation concerning the generalizability of findings. Although the initial recruitment sample comprised families experiencing at least one indicator of social or economic disadvantage, a greater proportion of vulnerable families were lost to attrition at school‐age follow‐up compared to less vulnerable families. Our findings may therefore be less applicable to families experiencing greater socioeconomic disadvantage.

### Implications for policy, practice, and future research

Effortful control is a critical skill for children's engagement in classroom learning and is predictive of long‐term school achievement (Diaz et al., 2017; Dindo et al., 2017). It has also been linked to better social functioning in peer and teacher relationships and improved mental health in later childhood and adolescence (Valiente et al., 2006; Wang et al., 2016). Importantly, it is a self‐regulatory skill that underlies children's resilience, enabling them to respond adaptively to challenges and adversity (Eisenberg et al., 2004). Accordingly, it should be a focal point for strengths‐based early parenting interventions.

Our findings highlight the potential benefits offered by intervention and prevention initiatives that are delivered in early childhood. Although we are unable to draw comparisons between early parenting programs and center‐ or school‐based programs, our findings nevertheless suggest that intervention during this stage of life is likely to have downstream benefits. Unlike center‐based or school‐based interventions, there is generally greater stability of context in the home environment and more opportunities for continued implementation of strategies, which may be one reason why persistent effects were observed in our study (Bailey et al., 2017). From a research perspective, our findings also illustrate the importance of conducting longer term follow‐ups to determine whether post‐intervention effects are sustained. Such follow‐ups are needed to accurately characterize costs and benefits of early intervention (Watts et al., 2019).

Finally, our findings with respect to the *smalltalk plus* condition suggest that the benefits of home coaching were essential for long‐term intervention effects. To effectively distribute resources and funds, further work is necessary to identify families who will benefit most from supplementary home coaching. Identifying elements of home coaching which are critical and those which are superfluous to promoting long‐term intervention effects should also be a priority for future research.

## CONCLUSIONS

In this study, we provide robust evidence that an early childhood parenting intervention, focused on improving the quality and frequency of parent–child interactions, produced later benefits for children's self‐regulation in the school years. Notably, these effects were observed for families that received the *smalltalk* playgroup intervention combined with home coaching, underscoring the importance of guided feedback and applied learning in achieving stronger outcomes from early parenting interventions. These findings indicate that parenting behaviors that scaffold children's regulatory responses and promote sustained interest and persistence in activities are important intervention targets for enhancing self‐regulation and wellbeing in later childhood.

## FUNDING INFORMATION

This research was funded by a National Health and Medical Research Council (NHMRC) Partnership Grant (GNT1076857) with partner funding from the Victorian Government Department of Education and Training. The collaboration was supported by the NHMRC Centre of Research Excellence in Child Language (GNT1023493). CB, EW, SB, JL, NH, and JN were supported by the Roberta Holmes Donation to La Trobe University.

## ETHICS STATEMENT

Ethics approval was granted by the La Trobe University Human Ethics Committee (No. 15–028), the Victorian Department of Education and Training (DET, formerly the Department of Education and Early Child Development) Research Committee, and the Catholic Education Offices of Ballarat, Melbourne, Sale, and Sandhurst. Parents provided written informed consent on behalf of themselves and the participating child. Teachers provided informed consent.

## Data Availability

The data and code necessary to reproduce the analyses presented in this paper are not publicly accessible. The analyses presented here were not preregistered.
